# Stopping Versus Continuing Metformin in Patients With Advanced CKD: A Nationwide Scottish Target Trial Emulation Study

**DOI:** 10.1053/j.ajkd.2024.08.012

**Published:** 2025-02

**Authors:** Emilie J. Lambourg, Edouard L. Fu, Stuart McGurnaghan, Bryan R. Conway, Neeraj Dhaun, Christopher H. Grant, Ewan R. Pearson, Patrick B. Mark, John Petrie, Helen Colhoun, Samira Bell, Scottish Diabetes Research Network Epidemiology Group, Rory McCrimmon, Catherine Armstrong, Alistair Emslie-Smith, Robert Lindsay, Sandra MacRury, John McKnight, Donald Pearson, Brian McKinstry

**Affiliations:** 1 Division of Population Health and Genomics, School of Medicine, University of Dundee, Dundee; 2 Institute of Genetics and Molecular Medicine, University of Edinburgh, Edinburgh; 3 School of Cardiovascular & Metabolic Health, College of Medical, Veterinary & Life Sciences, University of Glasgow, Glasgow, United Kingdom; 4 School of Health and Wellbeing, College of Medical, Veterinary & Life Sciences, University of Glasgow, Glasgow, United Kingdom; 5 Division of Pharmacoepidemiology and Pharmacoeconomics, Department of Medicine, Brigham and Women’s Hospital and Harvard Medical School, Boston, Massachusetts; 6 Department of Clinical Epidemiology, Leiden University Medical Center, Leiden, the Netherlands

**Keywords:** Chronic kidney disease, diabetes, epidemiology, metformin, target trial emulation

## Abstract

**Rationale & Objective:**

Despite a lack of supporting evidence, current guidance recommends against the use of metformin in people with advanced kidney impairment. This observational study compared the outcomes of patients with type 2 diabetes who continued versus stopped metformin after developing stage 4 chronic kidney disease (CKD) (estimated glomerular filtration rate [eGFR] < 30 mL/min/1.73 m^2^).

**Study Design:**

Nationwide observational cohort study.

**Setting & Participants:**

All adults with type 2 diabetes and incident stage 4 CKD in Scotland who were treated with metformin between January 2010 and April 2019.

**Exposure:**

Stopping versus continuing metformin within 6 months following incident stage 4 CKD.

**Outcome:**

Primary outcome was all-cause mortality. Secondary outcomes included major adverse cardiovascular events (MACE).

**Analytical Approach:**

Target trial emulation with clone-censor-weight design and marginal structural models fit for sensitivity analyses.

**Results:**

In a population of 371,742 Scottish residents with a diagnosis of type 2 diabetes before April 30, 2019, 4,278 were identified as prevalent metformin users with incident CKD stage 4. Within 6 months of developing CKD stage IV, 1,713 (40.1%) individuals discontinued metformin. Compared with continuing metformin, stopping metformin was associated with a lower 3-year survival (63.7% [95% CI, 60.9-66.6] vs 70.5% [95% CI, 68.0-73.0]; HR, 1.26 [95% CI, 1.10-1.44]), and the incidence of MACE was similar between both strategies (HR, 1.05 [95% CI, 0.88-1.26]). Marginal structural models confirmed the higher risk of all-cause mortality and similar risk of MACE in patients who stopped versus continued metformin (all-cause mortality: HR, 1.34 [95% CI, 1.08-1.67]; MACE: HR, 1.04 [95% CI, 0.81-1.33]).

**Limitations:**

Residual confounding.

**Conclusions:**

The continued use of metformin may be appropriate when eGFR falls below 30 mL/min/1.73 m^2^. Randomized controlled trials are needed to confirm these findings.

## Plain-Language Summary

Current guidance recommends against the use of metformin in people with advanced kidney impairment despite a lack of evidence. It is therefore currently unclear how the decision to stop versus continue metformin in patients who reach stage 4 CKD impacts their risk of mortality and cardiovascular events. This study showed that stopping metformin after reaching stage 4 CKD was associated with reduced survival that did not appear to be mediated by an increase in adverse cardiovascular outcomes. These findings may support the continued use of metformin in patients with advanced kidney impairment, but further research is needed to confirm these observations.

## Visual Abstract

**Figure:**
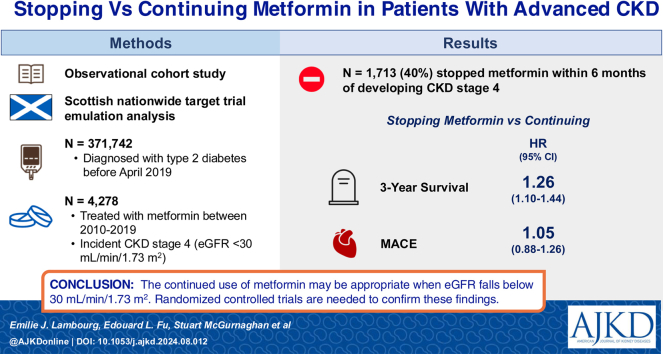

Editorial, p. 184

Diabetes is the leading cause of kidney failure globally with concurrent CKD stage 3 or greater (glomerular filtration rate [GFR] < 60 mL/min/1.73 m^2^) in a quarter of patients.^1^^,^^2^ Prescribing of glucose-lowering agents is challenging in patients with severely impaired kidney function (estimated GFR [eGFR] < 30 mL/min/1.73 m^2^), for whom sulfonylureas are most frequently prescribed (58%). Metformin undergoes rapid renal excretion without metabolism,^3^ and prescription rates are low in patients with reduced GFR due to the risk of lactic acidosis, a rare side effect caused by metformin accumulation.

Despite the lack of a clear causal association, current UK National Institute for Health and Care Excellence (NICE) guidelines state that metformin should neither be initiated nor continued in patients with eGFR < 30 mL/min/1.73 m^2^, a threshold below which metformin is considered unsafe.^4^ Current evidence regarding the benefits and safety of metformin in patients with CKD stage 4 remains limited, with no randomized controlled trial (RCT) comparing metformin with other antidiabetic agents in this population. However, in a pharmacokinetic study, Lalau et al^5^ demonstrated that a daily metformin dose of 500 mg appeared safe in patients with CKD stage 4 (eGFR < 30 mL/min/1.73 m^2^) with pharmacologically efficacious blood metformin concentrations and no cases of hyperlactatemia (>5 mmol/L) reported after 4 months of treatment. It is, however, important to note that this study only examined safety over a short time period and that data on longer term safety are lacking.

Our study used the target trial emulation framework and national routinely collected health care data to compare outcomes between patients who stopped versus continued metformin after reaching CKD stage 4. We hypothesized that the continuation of metformin in this context would not be associated with adverse outcomes.

## Methods

Additional information on methods is available in Item S1.

### Data Sources

A national retrospective observational study was performed using the previously described Scottish Diabetes Research Network–National Diabetes Study (SDRN-NDS) cohort. SDRN-NDS describes a data rich cohort of over 470,000 patients diagnosed with diabetes from 2006 to 2010 through linkage of multiple routine care databases.^6^ It is estimated to contain clinical information on over 99% of people with diabetes diagnosed in Scotland.^7^

### Target Trial

The target trial emulation framework aims to apply the design of a RCT to observational data, with the objective of improving the quality of observational studies and minimize preventable biases such as the prevalent user bias or the immortal time bias. This is a valuable tool when a trial is not feasible or practical and can be adapted to suit many causal questions where the objective is to examine the effect of an intervention. Recently, the target trial emulation approach has been increasingly applied in clinical research in various settings.^8^, ^9^, ^10^, ^11^, ^12^

The target trial framework involves explicit specification of the hypothetical trial one would conduct (eligibility criteria, treatment strategies, data analysis, etc) and then emulating it with observational data. One key aspect in target trial emulation is aligning eligibility criteria, treatment assignment, and start of follow-up, which removes preventable biases such as immortal time and depletion of susceptibilities bias.^13^^,^^14^ Note that the target trial framework does not mitigate the risk of unmeasured confounding. The full protocol of the pragmatic target trial and its emulation is available in Table S1 and Figure S2.

### Eligibility Criteria

We included all adults (≥18 years) with type 2 diabetes and incident CKD stage 4 between January 2010 and April 30, 2019, who were prevalent metformin users. Incident CKD stage 4 was defined as the presence of 2 eGFR values < 30 mL/min/1.73 m^2^ (calculated using the Chronic Kidney Disease Epidemiology Collaboration [CKD-EPI] 2009 equation^15^ assuming non-Black race) separated by a minimum of 90 days during the study period. A patient was considered a “prevalent metformin user” if they were prescribed metformin for more than 80% of the year before the index date (calculation of the medication possession ratio assuming a daily dose of 1,500 mg of metformin). Metformin discontinuation was defined as a gap of 90 days or more in metformin prescription records.

### Treatment Strategies

In this emulated target trial, we compared the following 2 treatment strategies:

1. Stop metformin within 6 months of reaching CKD stage 4.
2. Continue metformin for at least 6 months after reaching CKD stage 4.

Each study participant was duplicated (ie, virtually cloned), with each replicate assigned to 1 of the above-mentioned treatment strategies (1 assigned to stopping, the other to continuing), thereby making both study arms perfectly identical at baseline. At monthly intervals, we determined whether clones were adherent to their assigned treatment strategies. Those who deviated were artificially censored. Replicates assigned to the first strategy (“stop metformin within 6 months”) were censored at the end of the grace period had they not stopped metformin by that time.

Replicates assigned to the second strategy (“continue metformin for at least 6 months”) were censored if they stopped metformin before the end of the 6-month grace period, at the time they stopped taking their treatment. Artificial censoring due to deviation from the assigned treatment strategy is likely to be informative, thereby introducing selection bias, which is addressed by the use of inverse-probability-of-censoring weighting (IPCW). This differs from administrative censoring or censoring due to loss of follow-up where IPCW does not apply. Of note, an event only contributes to the arm in which the patient is still uncensored at the time of event, thereby controlling for immortal time bias. For instance, individuals who die within the grace period without stopping metformin have their replicates contributing follow-up time and a death event to both arms.

The choice of a 6-month grace period aimed to enable sufficient time for physicians to make the decision to stop metformin in a prevalent user. The number of individuals discontinuing metformin regularly decreased with time from the index date with an important drop around 6-7 months, explaining the choice for this cutoff point (Fig S1).

### Start and End of Follow-up

For each patient included in the cohort, the start of follow-up (time zero) was the date of the serum creatinine test corresponding to the second eGFR below 30 mL/min/1.73 m^2^. Follow-up continued until occurrence of an outcome of interest, loss to follow-up, administrative censoring (April 30, 2019), or 3 years, whichever came first.

### Study Outcomes

The primary outcome was all-cause mortality ascertained by NRS death records. Secondary outcomes were major adverse cardiovascular events (MACE), 3-year risk of cancer, and respiratory diseases–related death.

The relevant *International Classification of Diseases, Tenth Revision* (ICD-10) codes and positions searched for each outcome are documented in Table S2.

### Statistical Analyses

Baseline characteristics of the cohort were summarized as median and interquartile range for continuous variables that presented a skewed distribution and as mean ± standard deviation for continuous variables displaying a normal distribution. Categorical variables were presented as percentages.

Metformin use after the index date was inferred from pharmacy dispensation claims, assuming a daily dose of 1,000 mg. This was derived from the subgroup of patients for whom strength, number of pills, and directions were available (about 38% of the cohort). This enabled calculating the number of days of supply and defining when (if ever) treatment was discontinued (gap of 180 days or more).

Data were analyzed using the clone-censor-weight (CCW) method.^16^^,^^17^ The CCW method is implemented in 3 successive steps (Fig S3): first, each participant is cloned (duplicated) and assigned to a different strategy; then replicates are censored if and when they deviate from their assigned strategy; and finally, IPCW is used to adjust for the selection bias introduced by the censoring. Individual probabilities of remaining uncensored at each monthly interval were predicted using a pooled regression model with being uncensored as the outcome with time-fixed independent variables (age, sex, deprivation level, smoking status) and time-varying confounders including exposure to medications, comorbidities (cancer, heart diseases, etc), laboratory measurements (eGFR, hemoglobin A_1c_ [HbA_1c_]), blood pressure (systolic blood pressure and diastolic blood pressure), number of hospitalizations, as well as number of eGFR and HbA_1c_ measurements in the past year and diabetes duration. A separate model was fitted in each arm to capture potential treatment–covariates interactions. The weights are the inverse of these probabilities.

In the control arm (continue metformin), we cumulated probabilities over time while in the stopping arm there was only 1 possible time of event (censoring at 6 months if metformin has not been stopped by the end of the grace period); therefore, only 1 censoring probability was computed. Because individuals are present in both treatment strategies (due to the cloning step), there is no possible confounding at baseline. The covariate balance between the 2 arms was evaluated at the end of the grace period using the standardized mean difference (SMD) and plotted on a Love plot. Weighted cumulative incidence curves were estimated using a Kaplan-Meier estimator.

Outcome models included an indicator for time (integers representing the monthly intervals), treatment strategy, an interaction between them, and the weights estimated in the weight model. Additional statistical details are provided in Item S2.

### Sensitivity Analyses

In the sensitivity analyses, marginal structural models were fitted using stabilized weights to estimate the effect of time-varying metformin use on outcomes in the presence of time-dependent confounding. IPTW was used to adjust for time-varying confounding with the numerator and denominator built through separate pooled logistic regression models. These models include discontinuation as a dependent variable, and the numerator model includes an indicator for time and all time-fixed confounders as independent variables. The denominator model additionally includes time-varying confounders among the independent variables. The denominator aims to adjust for time-varying confounding while the numerator is added to stabilize the weights, thereby preventing them from becoming increasingly large.

Discontinuation was also defined as a period of 90 days or more free from metformin dispensation claims, and an assumption was made than once the treatment stopped it was not restarted (binary variable for discontinuation going from 0 to 1), thereby modeling time to discontinuation. This was permitted by the selection of a relatively long lag period for discontinuation after the estimated last day of supply and only a small proportion of patients restarting their treatment after stopping for 3 months or more.

Finally, the outcome model estimating the effect of metformin discontinuation is a weighted pooled logistic regression model that includes a binary time-dependent treatment variable for discontinuation, an indicator for time, and interactions between time and all time-fixed covariates. The outcome model was then used to estimate the adjusted cumulative incidence curves.

### Interpretation of Estimates

Marginal structural models aim to estimate the average causal effect of treatment discontinuation on outcomes in the original study population, provided the assumptions of exchangeability, positivity, consistency, and no model misspecification are met. This is complementary to but also contrasts with the CCW approach, which aims to estimate the average causal effect of treatment discontinuation during the first 6 months after reaching CKD stage 4 compared with a sustained treatment strategy for at least 6 months.

## Results

### Baseline Characteristics

**Figure 1:**
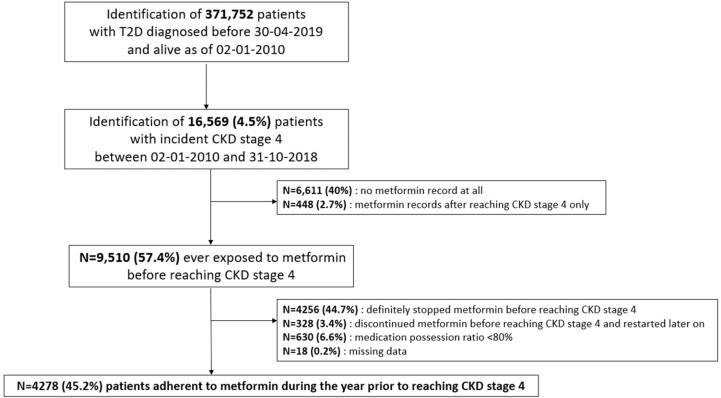
Study flow chart. Abbreviations: CKD, chronic kidney disease; T2D, type 2 diabetes.

Out of 371,742 Scottish residents with a diagnosis of type 2 diabetes before April 30, 2019, 4.5% (n = 16,569) reached CKD stage 4 during the study period. Of those, 4,278 (n = 25.8%) were adherent metformin users during the year prior to reaching CKD stage 4 and constituted the study cohort (Fig 1). This cohort was followed for a median time of 2.5 years (IQR, 1.2-4.2). The median age of the cohort was 77.0 years (IQR, 70.5-82.0), and 51% were women. The median eGFR at cohort entry was 27 (IQR, 24-29) mL/min/1.73 m^2^, and cardiovascular comorbidities were common, with ischemic heart disease and cerebrovascular diseases affecting 38.4% and 10.7%, respectively, of the cohort. (Table 1).

**Table 1:** Baseline Characteristics of the Cohort

Characteristics at Index Date	Values
**Demographics**	
Age, y	76.9 [70.5-82.0]
Age category	
<50	30 (1.2%)
50-70	971 (22.7%)
70+	3,277 (76.6%)
Women	2,194 (51.3%)
Smoking status	
Current smoker	413 (9.7%)
Ever smoker	1,891 (44.2%)
Never smoked	1,974 (46.1%)
Diabetes duration, y	14.0 [9.8-18.7]
**Laboratory Values**	
eGFR, mL/min/1.73 m^2^	27.0 [24.2-28.7]
HbA_1c_, mmol/mol	55.00 [47.5-66.0]
Total cholesterol, mmol/L	3.9 [3.3-4.5]
**Vitals**	
Systolic BP, mm Hg	136 [126-146]
Diastolic BP, mm Hg	71 [66-78]
Body mass index, kg/m^2^	30.5 [26.7-34.9]
**Comorbidities**	
Ischemic heart diseases	1,641 (38.4%)
Cerebrovascular diseases	457 (10.7%)
Other circulatory system diseases	1,031 (24.1%)
Cancer	732 (17.1%)
Chronic respiratory diseases	970 (22.7%)
Liver diseases	98 (2.3%)
**Ongoing Medications**	
ACE/ARB	3,564 (83.3%)
Thiazide diuretics	980 (22.9%)
α-Blockers	942 (22.0%)
Anticoagulants	3,098 (72.4%)
Lipid-regulating drugs	3,772 (88.2%)
Calcium-channel blockers	2,084 (48.7%)
Insulin	912 (21.3%)
Sulfonylurea	1,893 (44.2%)
DPP4 inhibitors	754 (17.6%)
GLP1-RA	170 (4.0%)
SGLT2 inhibitors	18 (0.4%)

Results are shown as median [interquartile range] for continuous variables, or as number of patients (percentage) for categorical variables. Abbreviations: ACE, angiotensin-converting enzyme; ARB, angiotensin receptor blocker; BP, blood pressure; DPP4, dipeptidyl peptidase 4; eGFR, estimated glomerular filtration rate; GLP1-RA, glucagon-like peptide 1 receptor agonists; HbA_1c_, hemoglobin A_1c_; SGLT2, sodium/glucose cotransporter 2.

### Metformin Treatment Trajectories

During the grace period (first 6 months of follow-up), 40% of the cohort (n = 1,713) stopped metformin. Of these, 4.3% (n = 73) restarted their treatment later during the follow-up period. Among those who continued taking metformin during the grace period, 54.8% (n = 2,344) discontinued later on during the follow-up period, and 44% continued taking metformin until the end of the follow-up period (Fig S2).

### Outcome Comparison

#### All-Cause Mortality and MACE

During the follow-up period, 1,702 individuals died, with the 3 most common causes of death being cardiovascular diseases (34.2%), cancer (17.4%), and respiratory diseases (10.1%), which includes both chronic lower respiratory diseases and pneumonia.

**Figure 2:**
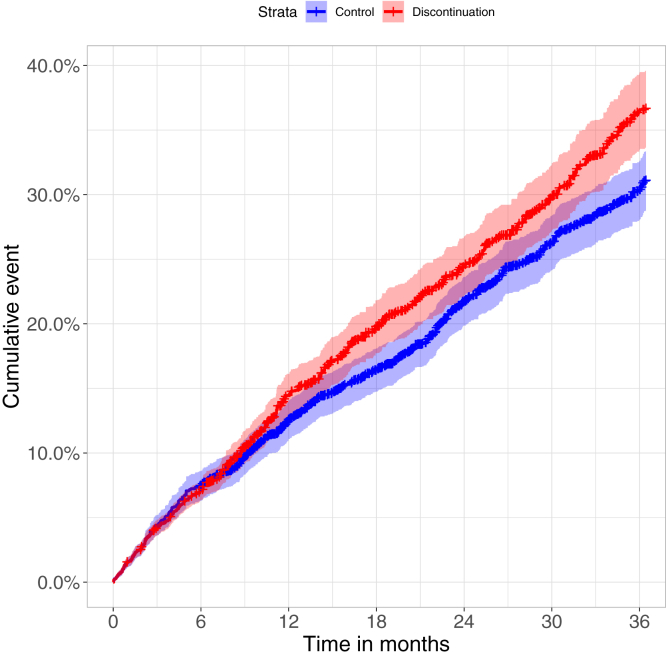
Weighted cumulative incidence curves for mortality, by treatment strategy.

We observed a higher 3-year survival (70.3% [95% CI, 67.9-72.8]) among those who continued metformin for at least 6 months, compared with those who stopped metformin within 6 months of reaching CKD stage 4 (63.4% [95% CI, 60.5-66.5]), after confounding adjustment (Fig 2). The corresponding hazard ratio (HR) was 1.23 (95% CI, 1.08-1.41) for stopping versus continuing. All covariates were adequately balanced at the end of the grace period (Fig S4; Table 2).

**Table 2:** Characteristics of Cloned Data Before and After Weighting at the End of the Grace Period

	Unweighted			Weighted		
	Continue (n = 2,344)	Discontinue (n = 1,627)	SMD	Continue (n = 4,045.1)	Discontinue (n = 3,877.43)	SMD
**Demographics**						
Age, y	76.25 ± 8.36	74.73 ± 9.20	0.174	75.62 ± 8.74	75.54 ± 8.88	0.009
Female	1,267 (54.1%)	785 (48.2%)	0.116	2,133.5 (52.7%)	2,021.3 (52.1%)	0.012
Diabetes duration, y	15.27 ± 7.13	15.14 ± 6.93	0.019	15.29 ± 7.01	15.30 ± 7.06	0.002
Smoking status			0.1			0.046
Current smoker	194 (8.3%)	183 (11.2%)		366.7 (9.1%)	367.5 (9.5%)	
Ever smoked	1,043 (44.5%)	706 (43.4%)		1,893.2 (46.8%)	1,725.8 (44.5%)	
Never smoked	1,107 (47.2%)	738 (45.4%)		1,785.3 (44.1%)	1,784.1 (46.0%)	
**Laboratory Measurements**						
eGFR, mL/min/1.73 m^2^	31.29 ± 8.55	27.96 ± 8.50	0.391	30.11 ± 9.42	29.84 ± 8.48	0.03
HbA_1c_, mmol/mol	59.49 ± 17.79	64.85 ± 21.84	0.269	60.80 ± 18.72	62.98 ± 21.27	0.109
**Vitals**						
SBP, mm Hg	136.26 ± 18.59	136.91 ± 19.35	0.034	136.84 ± 19.22	136.46 ± 19.12	0.02
DBP, mm Hg	71.46 ± 10.24	72.09 ± 10.83	0.06	71.70 ± 10.24	71.93 ± 10.55	0.022
BMI, kg/m^2^	31.34 ± 6.44	31.14 ± 6.56	0.03	31.09 ± 6.33	31.34 ± 6.55	0.039
**Comorbidities**						
Ischemic heart diseases	917 (39.1%)	658 (40.4%)	0.027	1,587.3 (39.2%)	1,560.1 (40.2%)	0.02
Cerebrovascular diseases	258 (11.0%)	203 (12.5%)	0.046	452.8 (11.2%)	489.9 (12.6%)	0.044
Other circulatory system diseases	574 (24.5%)	442 (27.2%)	0.061	1,091.0 (27.0%)	976.1 (25.2%)	0.041
Chronic lower respiratory diseases	518 (22.1%)	458 (28.1%)	0.14	995.7 (24.6%)	993.6 (25.6%)	0.023
Cancer	407 (17.4%)	293 (18.0%)	0.017	680.0 (16.8%)	687.1 (17.7%)	0.024
**Medications**						
Anticoagulants	1,596 (68.1%)	1,068 (65.6%)	0.052	2,670.4 (66.0%)	2,588.9 (66.8%)	0.016
ACE/ARB	1,603 (68.4%)	805 (49.5%)	0.392	2,395.6 (59.2%)	2,318.6 (59.8%)	0.012
α-Blockers	440 (18.8%)	326 (20.0%)	0.032	759.4 (18.8%)	746.4 (19.2%)	0.012
Lipid-regulating drugs	1,912 (81.6%)	1,181 (72.6%)	0.215	3,081.3 (76.2%)	2,970.8 (76.6%)	0.01
Calcium-channel blockers	963 (41.1%)	642 (39.5%)	0.033	1,651.7 (40.8%)	1,566.0 (40.4%)	0.009
Thiazide diuretics	354 (15.1%)	145 (8.9%)	0.191	501.7 (12.4%)	492.0 (12.7%)	0.009
Sulfonylurea	900 (38.4%)	742 (45.6%)	0.146	1,736.8 (42.9%)	1,607.4 (41.5%)	0.03
Insulin	496 (21.2%)	483 (29.7%)	0.197	936.3 (23.1%)	1,005.1 (25.9%)	0.065
DPP4 inhibitors	357 (15.2%)	330 (20.3%)	0.132	639.1 (15.8%)	665.2 (17.2%)	0.037
**Health Care Encounters in Past Year**						
No. of SBP measurements	3.73 ± 3.09	4.60 ± 3.64	0.256	4.14 ± 3.38	4.03 ± 3.31	0.032
No. of hospitalizations	1.47 ± 2.45	2.84 ± 3.68	0.439	2.05 ± 2.96	2.16 ± 3.17	0.036
No. of eGFR	6.48 ± 4.84	9.52 ± 6.23	0.545	7.66 ± 5.44	7.95 ± 5.76	0.052
No. of HbA_1c_ measurements	2.32 ± 1.42	3.07 ± 1.86	0.457	2.64 ± 1.70	2.70 ± 1.66	0.036

Values for continuous variables given as mean ± SD and for categorical variables as count (percentage). Abbreviations: ACE, angiotensin-converting enzyme; ARB, angiotensin receptor blocker; BMI, body mass index; DBP, diastolic blood pressure; DPP4, dipeptidyl peptidase 4; eGFR, estimated glomerular filtration rate; HbA_1c_, hemoglobin A_1c_; SBP, systolic blood pressure; SMD, standardized mean difference.

During the 3-year follow-up period, 915 MACE were recorded. Stopping metformin was not associated with the risk of MACE, with a HR of 1.05 (95% CI 0.88-1.26) (Fig S5).

#### Other Outcomes

During the follow-up period, 296 individuals (17.4%) died from cancer, and 172 (10.1%) died from respiratory diseases. Discontinuing metformin was associated with a higher risk of death from respiratory diseases (HR, 1.51 [95% CI, 1.06-2.12]). There was, however, no association between stopping or continuing metformin and risk of cancer (HR, 1.07 [95% CI, 0.80-1.44]).

#### Sensitivity Analyses

Using a marginal structural model, the HR for all-cause mortality was 1.34 (1.08-1.67), and for MACE was 1.04 (0.81-1.33).

## Discussion

In this nationwide observational study, 40% of people with type 2 diabetes regularly treated with metformin stopped their treatment within 6 months of reaching CKD stage 4. Using target trial emulation with the CCW method, early stopping of metformin after reaching CKD stage 4 was associated with a modest increase in all-cause mortality (HR, 1.23 [95% CI, 1.08-1.41]), but no difference in risk of major cardiovascular events (HR, 1.05 [95% CI, 0.88-1.26]). Investigation of secondary outcomes showed that the risk of death from respiratory diseases, which included lower respiratory tract infections, was higher in patients who discontinued metformin (HR, 1.51 [95% CI, 1.06-2.12]). There were no differences between the 2 arms with regards to cancer-related mortality (HR, 1.07 [95% CI, 0.80-1.44]).

Recently, Yang et al^18^ also investigated outcomes associated with metformin discontinuation in patients reaching CKD stage 4. Their results on all-cause and pneumonia-related mortality align with ours as they reported an increased risk in those who discontinued their treatment (HR, 1.22 [95% CI, 1.18-1.27], and HR, 1.06 [95% CI, 1.00-1.13], respectively). However, they also found an increased risk of MACE (HR, 1.40 [95% CI, 1.29-1.52]).

Current guidelines still contraindicate metformin in patients with CKD stage 4 but face increasing controversy due to recent studies suggesting that cautious use of metformin in this group may prove safe and beneficial, provided adequate dose-adjustment (500 mg per day according to Lalau et al^5^) and appropriate monitoring are implemented.^18^^,^^19^ As such, clinical practice is variable.^20^ Two studies from the United States estimated that about 10% of individuals with an eGFR below 30 mL/min/1.73 m^2^ were exposed to metformin.^21^^,^^22^ Similarly, in lower income countries, metformin is commonly used in patients with CKD stage 3 and 4.^23^

We found that continuing metformin for at least 6 months after reaching CKD stage 4 was associated with a lower risk of death (stopping vs continuing HR, 1.23 [95% CI, 1.08-1.41]) that did not appear to be mediated by a lower cardiovascular risk (HR, 1.05 [95% CI, 0.88-1.26]). In the general population and in patients with CKD stage 3,^24^^,^^25^ metformin use has been associated with lower risks of both all-cause mortality and cardiovascular death.

The cardioprotective effects of metformin are likely related to decreases in body mass index and glucose/HbA_1c_ levels, but also improvements in blood pressure control and dyslipidemia. However, this pattern may be different in patients with later CKD stages. Indeed, a systematic review and meta-analysis of observational studies including 3 post hoc analyses of RCTs demonstrated that metformin use was associated with significant lower risks of all-cause mortality (pooled risk ratio [RR], 0.71 [95% CI, 0.61-0.84]) and cardiovascular death (pooled RR, 0.76 [95% CI, 0.60-0.97]) in patients with CKD stages 1-3, but there was no significant relationship in individuals with CKD stages 4-5 (pooled RR for all-cause mortality, 0.80 [95% CI, 0.49-1.31]; pooled RR for cardiovascular events, 0.94 [95% CI, 0.68-1.30]).^25^ Another Korean study conducted among 97,713 patients with diabetes and eGFR below 60 mL/min/1.73 m^2^ even suggested a significantly increased risk of MACE among metformin-users compared with nonusers (HR, 1.20 [95% CI, 1.14-1.26]), despite concomitantly reporting a decreased risk of all-cause mortality and end-stage kidney disease.^26^

The reasons why the cardiovascular benefits of metformin may not extend to patients with later CKD stages remain unclear. It is possible that in these patients the etiology of CKD may be multifactorial with a nontraditional risk factor profile, which is less amenable to the benefits of metformin treatment. Another hypothesis is that individuals included in our study may already be at particularly high cardiovascular risk, with 38% of our cohort experiencing prior ischemic heart disease, 83% and 88% receiving angiotensin-converting enzyme/angiotensin receptor blocker and lipid-lowering agents, respectively, and 72% being treated with anticoagulants. Post hoc analyses of the SAVOR-TIMI 53 Trial, which included patients with type 2 diabetes and high cardiovascular risk, suggested that exposure to metformin had no impact on mortality or cardiovascular events in the subgroup of participants with GFR less than 45 mL/min/m^3^. The findings were at significant risk of prevalent user bias because metformin use was dichotomized as never versus ever on trial inception.^27^ Finally, a large proportion of our cohort (44.2%) was concomitantly treated with sulfonylureas, which have been associated with deleterious cardiovascular outcomes.^28^ It is possible that the extensive use of sulfonylureas may counterbalance the potential cardioprotective benefits of metformin, resulting in a null effect.

When examining additional outcomes, we observed a significantly lower risk of death from lower respiratory disease in those who continued metformin. Consistent with this finding, a propensity-matched cohort of around 50,000 individuals with type 2 diabetes revealed that metformin users had lower rates of bacterial pneumonia (HR, 0.89 [95% CI, 0.84-0.94]), invasive mechanical ventilation (HR, 0.77 [95% CI, 0.73-0.82]), and respiratory-related death (HR, 0.64 [95% CI, 0.56-0.74]). The risks of respiratory outcomes were further reduced as cumulative exposure to metformin increased.^29^ Wiernsperger et al^30^ reviewed how metformin interferes with the pathophysiological mechanisms implicated in SARS-CoV-2 infection, independently from diabetes. They suggest that metformin’s pleiotropic profile could improve outcomes of patients with COVID-19 through reduction of inflammation, unique microcirculatory protective effects, but also protection from the cytokine storm, decrease of virus entry into cells, and prevention of secondary fibrosis.

Our study has several strengths. The cohort was created using a national diabetes registry capturing over 99% of individuals with a diabetes diagnosis in Scotland. Compared with prescription data, the access to pharmacy dispensation claims reduced the risk of misclassification bias with regards to metformin exposure. Furthermore, our use of a target trial emulation design limits the risk of prevalent user and immortal time bias that is common to many traditional observational studies.

Limitations include the potential for residual confounding, which cannot be excluded despite the application of the target trial framework, which only promotes good practices in designing observational studies. Similarly, the CCW method removes all confounding at baseline only and further adjusts for measured confounding through IPCW, but it does have the ability to account for unmeasured confounding. Misclassification bias may have occurred in relation to the ascertainment of CKD stage 4 and determination of an index date referred to as the date of “reaching CKD stage 4.” The latter was based on a second eGFR reading below 30 within the study period (separated by at least 90 days from the first one), which may have been inaccurate in some cases if resulting from 2 successive episodes of acute kidney injury. Nevertheless, this algorithm has been validated previously for CKD stage 3 or more with sensitivity, specificity, and diagnostic accuracy of 99%, 99%, and 98%, respectively.^31^

Our cohort is likely to be representative of the UK population with diabetes but may not be generalizable to populations of different ethnicities or in countries without universal health coverage. Furthermore, the metformin prescribing data were obtained from pharmacy dispensation, so we are unable to ascertain adherence. In addition, determination of the daily dose of metformin prescribed (1,500 mg before the index date, 1,000 mg after) relies on an assumption inferred from the average calculated in the subgroup of the cohort for whom directions were available and does not account for different dosage regimen among patients.

In summary, we demonstrated that continuing metformin for at least 6 months following a confirmed eGFR drop below 30 mL/min/1.73 m^2^ was associated with a small but significant decrease in all-cause mortality that did not appear to be mediated by cardioprotective effects. Further studies, including RCTs, are warranted to confirm those results and establish which mechanism could explain the reduction in all-cause mortality associated with exposure to metformin in this population.

## Supplementary Material

Supplementary File (PDF) Figures S1-S5. Items S1-S2. Tables S1-S2.
