# Supplementary material for: Stopping Versus Continuing Metformin in Patients With Advanced CKD: A Nationwide Scottish Target Trial Emulation Study
Source: Am J Kidney Dis. 2025 Feb;85(2):196–204.e1. doi: 10.1053/j.ajkd.2024.08.012 (PMC12101959; doi:10.1053/j.ajkd.2024.08.012)
Supplement: Supplementary File (PDF) — Figures S1-S5. Items S1-S2. Tables S1-S2. [file mmc1.pdf]

Figure S1: Pattern of metformin discontinuation during study follow-up

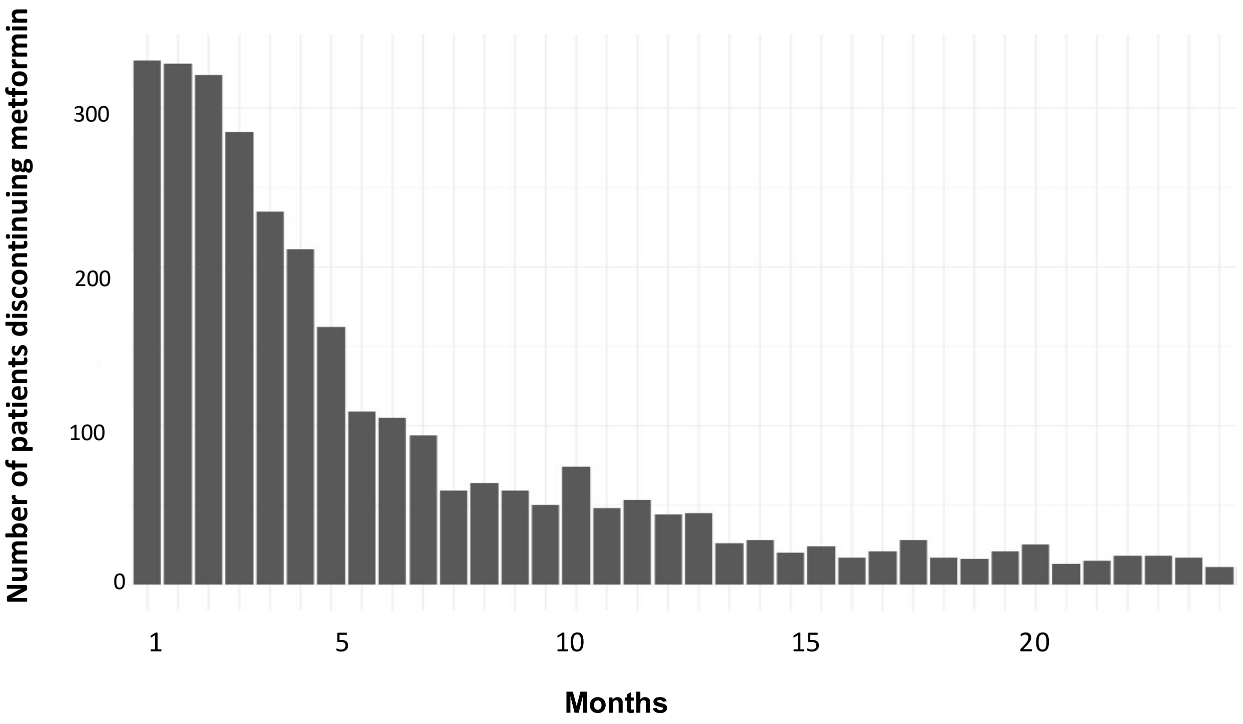

Figure S2: Sankey Diagram

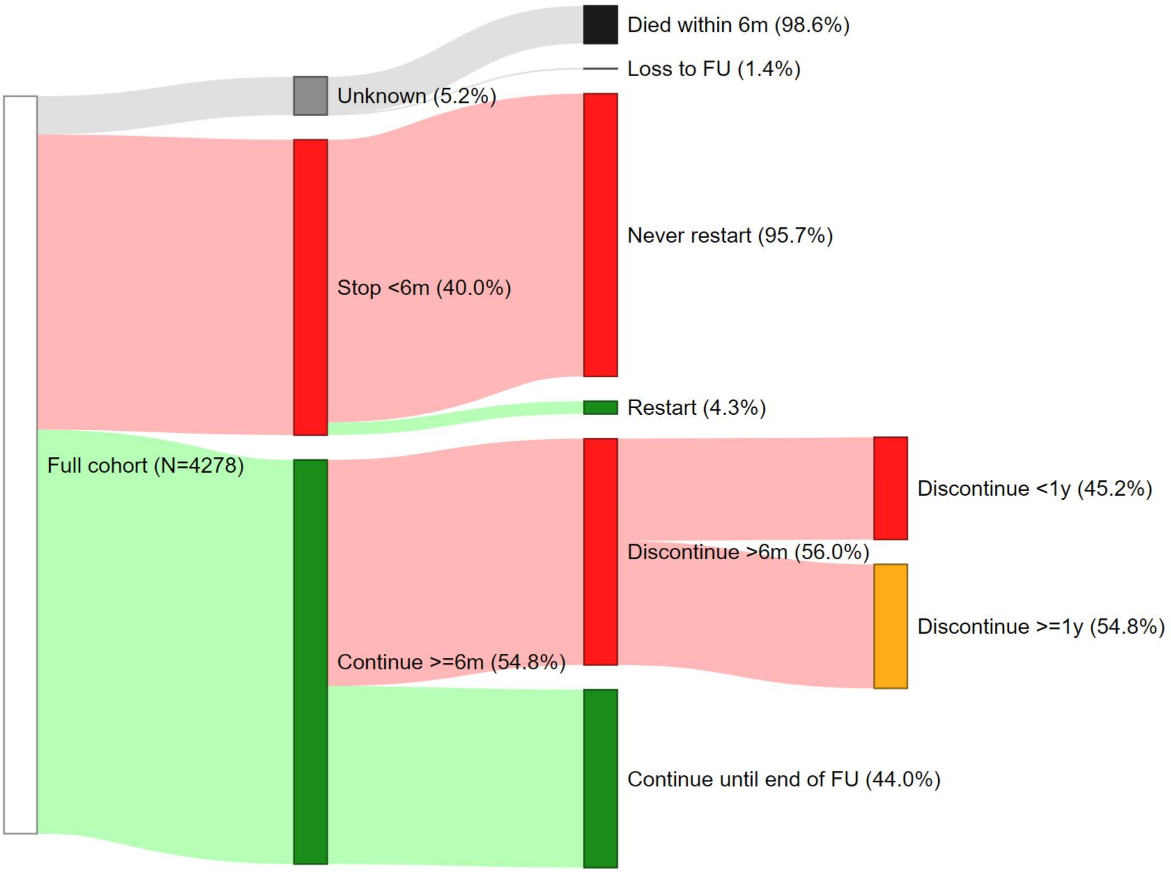

Figure S3: Three steps of the clone-censor-weight method.

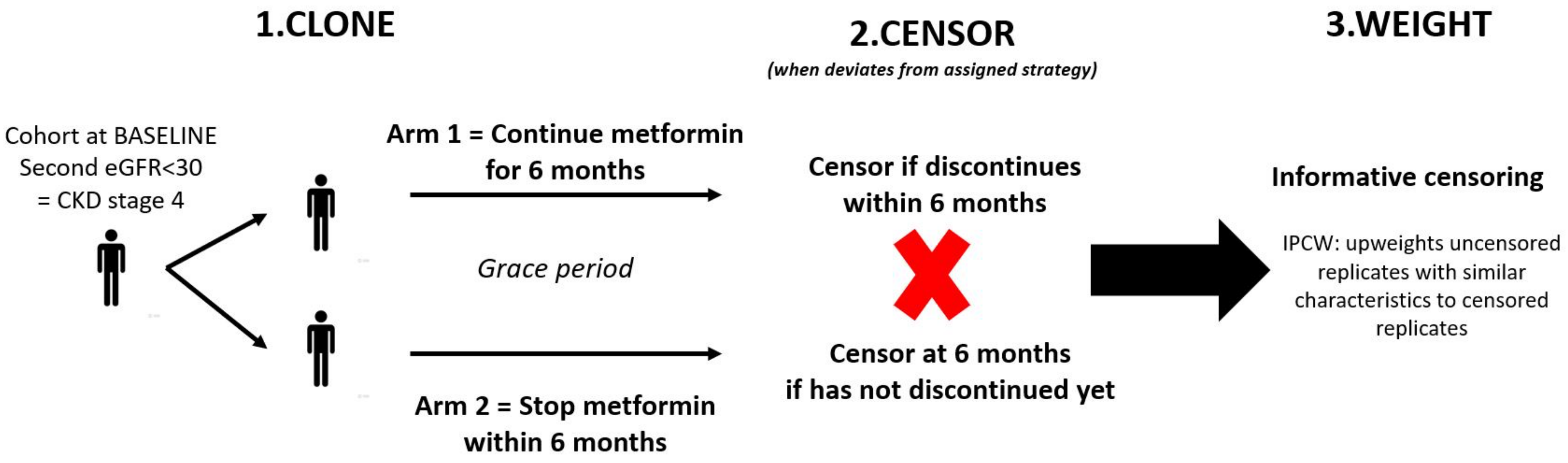

Obtaining unbiased estimates using the clone-censor-weight method requires that models used to calculate the weights are correctly specified as well as verification of the assumptions of positivity, consistency and no unmeasured confounders.

Covariate balance

To evaluate the ability of our model to generate weights removing covariate imbalance between the two arms, the standardised mean difference (SMD) was calculated for all confounders included. The SMD is defined for each covariate as the weighted mean difference between the two arms divided by the weighted pooled standard deviation. A variable with SMD above 10% is considered imbalanced and may suggest a misspecification of the weight model [1].

Outcome model

The outcome model was a weighted pooled logistic regression model fitted on the stacked datasets (one dataset per arm), to estimate the intention-to-treat effect of continuing versus stopping metformin within 6 months of reaching CKD stage 4. The weights were those estimated using the IPCW methodology detailed previously. Weighted cumulative incidence curves illustrated the differential effect of each treatment strategy on outcomes. In order to account for the weighting process, 95% confidence intervals were computed using nonparametric bootstrap with 500 samples.

Marginal Structural Models

A weighted Cox model – or Marginal Structural Model – was another method to estimate the effect of time-varying metformin use on outcomes in presence of time-dependent confounding.

Inverse probability of treatment weights were generated, creating a “pseudopopulation” in which there was no confounding (based on our panel of measured confounders). To create stabilised weights, the main step was to fit separate logistic regression models for the numerator and denominator. Both models had “metformin discontinuation” as outcome. Independent variables included an indicator of time and time-fixed covariates for the numerator, to which were added all time-varying covariates for the denominator model.

Finally, a weighted Cox model estimated the effect of discontinuing versus continuing metformin (outcome model). All baseline covariates were reincluded since the use of stabilised weights improves the variance but does not fully account for baseline covariates (present in both numerator and denominator), therefore, these must be readjusted for in the final model.

Ethical approval

NHS Data governance rules do not permit us to secondarily share the data directly. However, Bone fide researchers can apply to the Scottish Public Benefits and Privacy Protection Committee for access to these data. This research was conducted with approval from the Public Benefit Privacy Protection Panel (PBPP ref. 1617- 0147) with approval from

the Scotland A Research Ethics Committee (ref. 11/AL/0225). All datasets were de-identified before analysis.

Figure S4: Love plot depicting covariate balance between the two treatment arms before (in red) and after (in blue) weighting

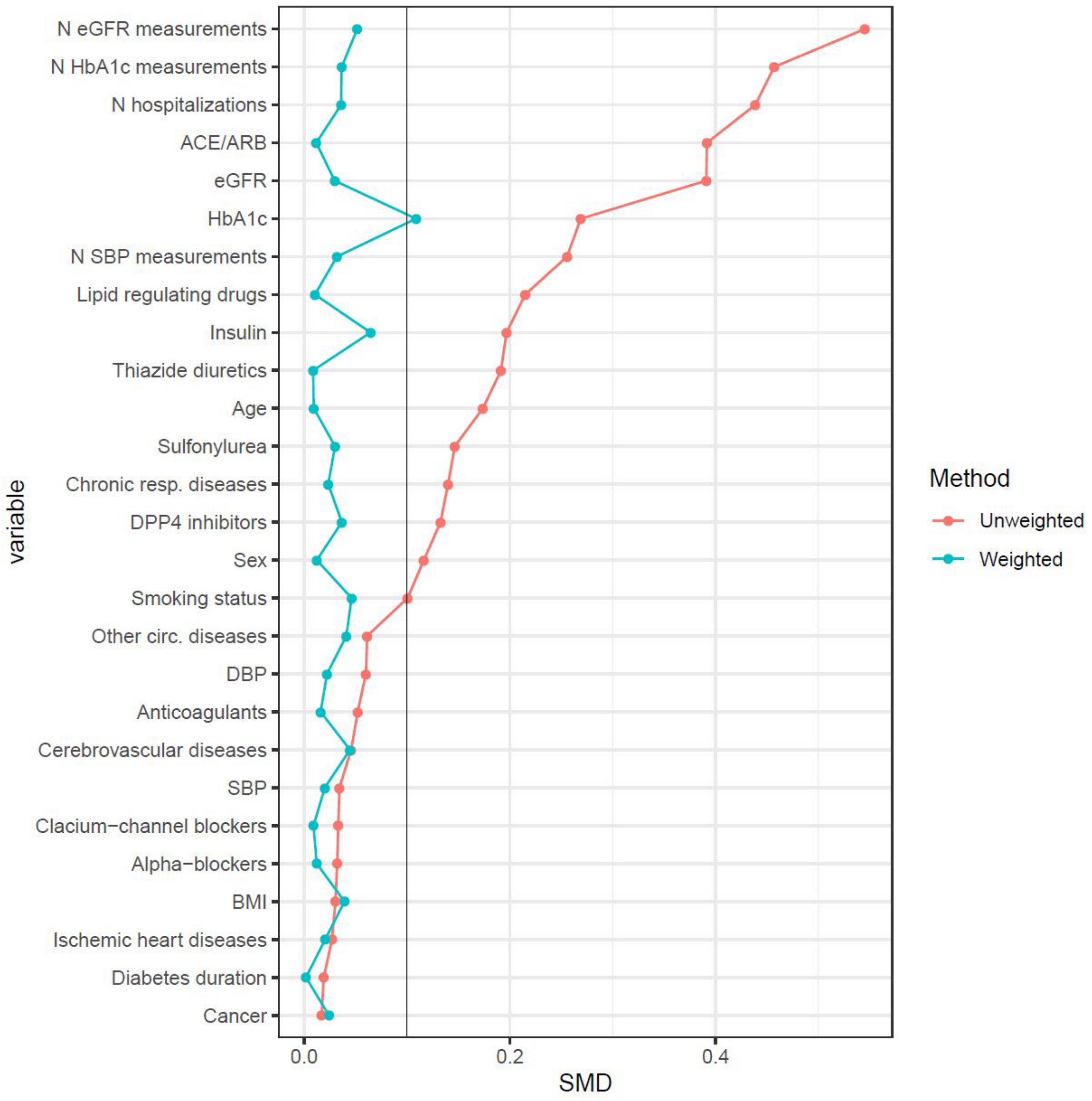

Figure S5: Weighted cumulative incidence curves for MACE, by treatment strategy

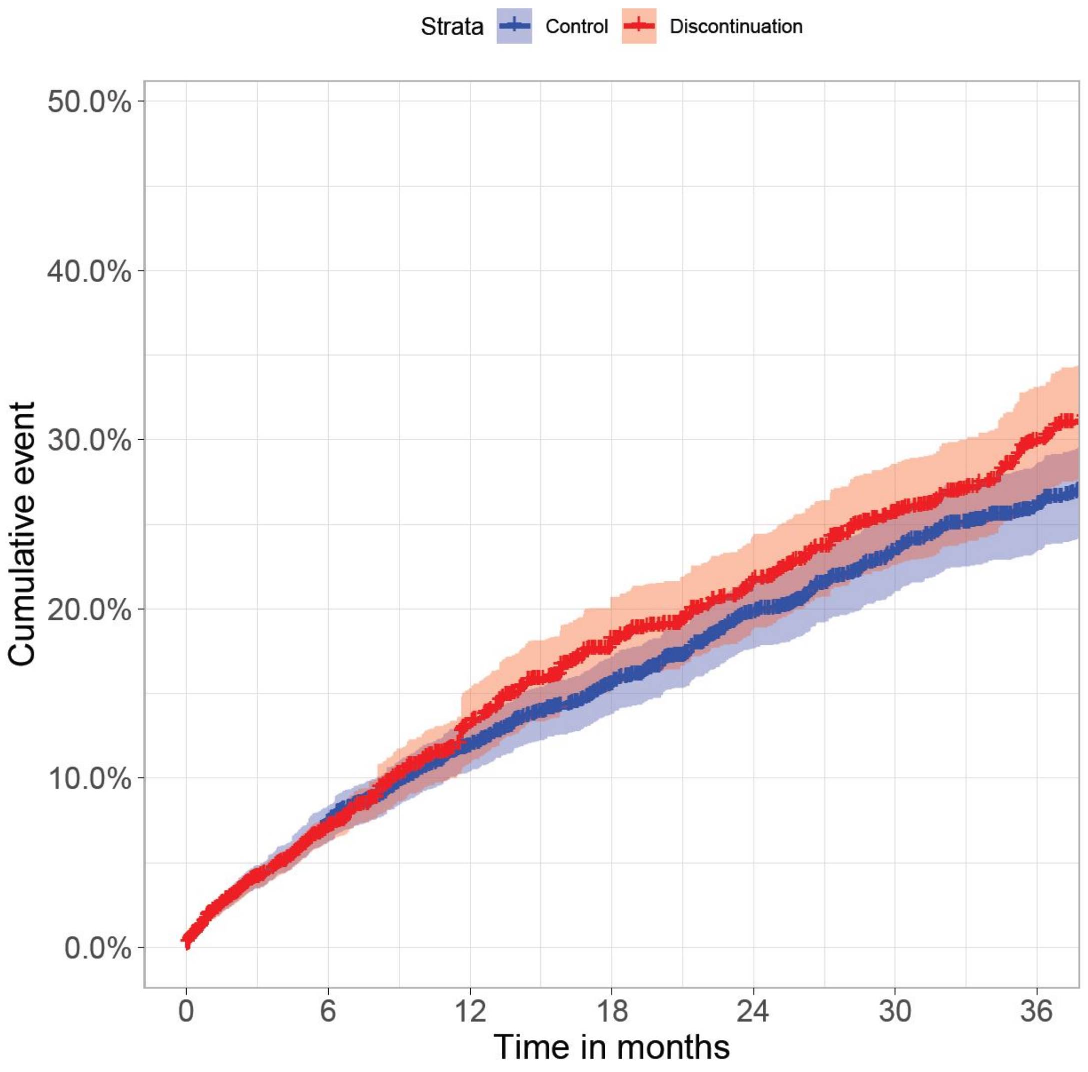

Item S1: Methods

Data sources: Diagnosis of diabetes was established using the Scottish Care Information-Diabetes Collaboration (SCI-DC), a high-quality integrated database used for routine diabetes care nationally The SCI-DC database is retrospectively and prospectively updated in real time from primary and secondary care systems including relevant prescription medications and laboratory test results constituting a diabetes registry for Scotland [2]. Data were linked using the Community Health Index number (a national unique health record identifier) with other administrative and healthcare datasets including the Scottish Renal Registry (SRR), Scottish Morbidity Records (SMR01) and National Records of Scotland (NRS). The SRR is a national registry of patients receiving kidney replacement therapy for established kidney failure with complete data coverage [3]. SMR01 records hospital episode data for every patient discharged following inpatient or day case care from an NHS Scotland bed including ICD-10 coded primary and secondary diagnoses [4]. NRS captures data on every person who has died in Scotland since 1985 with cause(s) of death coded according to ICD-10 [5]. Data were deidentified and uploaded to a unique safe haven environment for analyses [2].

Eligibility criteria: Patients with a history of chronic kidney replacement therapy (KRT, dialysis or a kidney transplant) were excluded as were those who discontinued metformin before reaching CKD stage 4.

Treatment strategies: We chose to examine an intention-to-treat effect (disregarding treatment patterns after the initial six months), as only a small proportion of patients (<5%) restarted metformin following discontinuation during the grace period.

Ethical approval: Approval for this work was obtained from the Public Benefit Privacy Protection Panel (PBPP ref. 1617- 0147) with approval from the Scotland A Research Ethics Committee (ref. 11/AL/0225). All datasets were de-identified before analysis.

Outcomes: MACE was a composite of fatal and non-fatal cardiovascular events defined as ischaemic heart diseases, myocardial infarction, cerebrovascular diseases and cardiac arrest determined from ICD-10 codes from SMR01 and NRS. Other secondary outcomes were obtained from NRS causes of death.

Item S2: Statistical analyses

Ever/never presence of diagnosis code was used or, in the case of continuous variables, the last value was carried forward. Patients who did not have any measurement recorded prior to their index date for one of the continuous variables adjusted for were excluded.

The clone-censor-weight method

Three successive steps are required to emulate a trial using the clone-censor-weight method [6]:

1) **Cloning:** at the index date (second eGFR below 30 ml/min/1.73 m^2 during the study period), each eligible patient was duplicated (cloned), and each replicate was assigned to one of the two treatment strategy (discontinue metformin within 6 months or continue for at least 6 months). Following the implementation of the cloning step, the original dataset became twice as large, as it contained two exact copies of each patient.

2) **Censoring**: At monthly intervals, we determined whether clones were adherent to their assigned treatment strategies. Those who deviated were artificially censored. Replicates assigned to the strategy “continue metformin for at least 6 months” were censored if and when they stopped metformin before the end of the 6-month grace period. Replicates assigned to the strategy “stop metformin within 6 months” were censored at the end of the grace period, had they not stopped metformin by that time.

3) **Weighting**: Censoring clones who are not adherent anymore to their assigned treatment strategy is likely to be informative, thereby introducing selection bias. This issue can be addressed by the use of Inverse-Probability-of-Censoring Weighting (IPCW), which upweights uncensored replicates remaining in the risk set that present similar characteristics to censored replicates. IPC weights are calculated using a pooled logistic regression model with the probability of remaining uncensored as outcome. Independent variables included time-fixed and time-varying confounders as well as a function of time. Pooled logistic regression models were fitted separately in each arm to account for differences in censoring pattern and better capture treatment by covariate interaction [7]. Confounders adjusted for included: demographic variables (age, sex, deprivation level, etc), smoking status, exposure to medications, comorbidities (cancer, heart diseases, etc), laboratory measurements (eGFR, Hba1c), blood pressure (SBP and DBP), number of hospitalisation as well as number of eGFR and HbA1c measurements in the past year, diabetes duration.

Table S1: Emulated Trial Protocol

Protocol component	Description of target trial	Description of emulation
Eligibility criteria	***Inclusion criteria:***<br>Patients with type 2 diabetes, prevalent metformin users, with incident CKD stage 4/5 between January 2010 and April 30th, 2019.<br><br>***Exclusion criteria:***<br>1. Patients on dialysis or kidney transplant recipients<br>2. < 18 years old	***Inclusion criteria:***<br>Patients developing CKD stage 4/5 (2 eGFR <30mL/min/1.73m2 separated by at least 90 days) adherent to metformin (>80% of the year prior to reaching CKD stage 4 covered by metformin prescriptions) between January 15th, 2010 and October 30th, 2018.<br>***Exclusion criteria:***<br>1. Patients on dialysis or kidney transplant recipients<br>2. < 18 years old<br>3. Missing baseline data
Treatment strategies	**Intention-To-Treat (ITT)**<br>1. Continue metformin within 6 months of reaching CKD stage 4<br>2. Stop metformin within 6 months of reaching CKD stage 4	**ITT**: Same as target trial.
Assignment procedures	Patients were randomized to either treatment strategy at baseline and were aware of their assigned strategy	Randomization is emulated by cloning each individual and then assigning each clone to one of the treatment strategies.
Follow-up	Follow-up started at the assignment of a strategy and ended at the earliest of outcomes of interest, death, loss-to-follow-up, administrative censoring (April 30th, 2019) or 3 years, whichever came first.	Same as target trial. Follow-up and assignment start at the sampling date of the second observed eGFR<30mL/min/1.73m2 during the study period
Outcome	***Primary outcomes:***<br>• All-cause mortality.<br>• MACE= composite of fatal/non-fatal cardiovascular events. CV events defined as a combination of ICD-10	All-cause mortality is identified from the National Records of Scotland data (where causes of death are ICD-10 coded).<br><br>Cardiovascular events are defined as a combination of ICD-10 codes for

	codes for ischemic heart diseases and stroke.<br>***Secondary outcomes:***<br>• cancer-related death<br>• respiratory diseases-related death	ischemic heart diseases, myocardial infarction and stroke.<br><br>Secondary outcomes are identified from relevant ICD-10 codes in SMR01 data.
Causal contrast	Intention-to-treat effect	Same as target trial.
Estimands	Hazard ratio<br>3-year survival<br>Cumulative incidence functions for cause-specific outcomes	Same as target trial.
Analysis plan	Intention-to-treat analysis; Individuals are artificially censored when they deviate from their assigned strategy as follows:<br>*Stop within 6 months (and ignore what happens after that):* Censor at month 6 if treatment has not yet been discontinued.<br>*Continue within 6 months:* Censored if individual stopped metformin during the first 6 months after second eGFR<30.<br><br>Note that inverse probability weighting is required also in a randomized trial to validly estimate the per-protocol effect.	Same as target trial, except the intention-to-treat analysis is conducted in an expanded data set that includes two clones for each eligible patient (one for each treatment strategy).<br><br>Inverse probability of censoring (IPC) weights are estimated as a function of time-fixed and time dependent variables. Variables include:<br>**Time-fixed only**: age, sex, diabetes duration in years, smoking status<br>**Time-fixed and time-varying**: eGFR, Hba1c, SBP, DBP, BMI, comorbidities (ischemic heart diseases, other heart diseases, stroke, other circulatory system diseases, cancer), medications (ACE/ARB, statins, diuretics including thiazide diuretics and sulfonylureas, antihypertensor alpha adrenoreceptors antagonists, calcium channel blockers anticoagulants, insulin, sulfonylurea, DPP4), rolling number of eGFR measurements in the past year, rolling number of HBA1c measurements in the past year, rolling number of hospitalizations in the past year

Table S2: ICD-10 codes for primary and secondary outcomes

Outcome	ICD-10 code	Dataset and position
**MACE**	I20-I25, I46, I60-I69, G45, G46	First 2 positions of NRS and SMR01
**Cancer-related mortality**	All C codes	First 2 positions of NRS
**Mortality related to respiratory diseases**	J00-J99	First 2 positions of NRS

References

1. Maringe, C., Benitez Majano, S., Exarchakou, A., Smith, M., Rachet, B., Belot, A., & Leyrat, C. (2020). Reflection on modern methods: trial emulation in the presence of immortal-time bias. Assessing the benefit of major surgery for elderly lung cancer patients using observational data. *Int J Epidemiol,* 49(5), 1719-1729. doi:10.1093/ije/dyaa057
2. McGurnaghan SJ, Blackbourn LAK, Caparrotta TM, Mellor J, Barnett A, Collier A, et al. Cohort profile: the Scottish Diabetes Research Network national diabetes cohort - a population-based cohort of people with diabetes in Scotland. *BMJ Open.* 2022;12(10):e063046.
3. Simpson K. The Scottish renal registry. *Scottish medical journal*. 1993;38(4):107-9.
4. Public Health Scotland. General Acute Inpatient and Day Case - Scottish Morbidity Record (SMR01) 2020. https://publichealthscotland.scot/services/national-data-catalogue/national-datasets/search-the-datasets/general-acute-inpatient-and-day-case-scottish-morbidity-record-smr01/
5. National Records of Scotland. Statutory Register of Deaths. https://www.nrscotland.gov.uk/research/guides/statutory-registers/deaths
6. Hernán, M. A. (2018). How to estimate the effect of treatment duration on survival outcomes using observational data. *BMJ*. 2018;360:k182. doi:10.1136/bmj.k182
7. Fu, E. L., Evans, M., Clase, C. M., Tomlinson, L. A., van Diepen, M., Dekker, F. W., & Carrero, J. J. Stopping renin-angiotensin system inhibitors in patients with advanced CKD and risk of adverse outcomes: a nationwide study. *Journal of the American Society of Nephrology.* 2021;32(2):424. doi: 10.1681/ASN.2020050682.
